# Chemical Characterisation of the Coarse and Fine Particulate Matter in the Environment of an Underground Railway System: Cytotoxic Effects and Oxidative Stress—A Preliminary Study

**DOI:** 10.3390/ijerph120404031

**Published:** 2015-04-13

**Authors:** Anna Maria Spagnolo, Gianluca Ottria, Fernanda Perdelli, Maria Luisa Cristina

**Affiliations:** Department of Health Sciences, University of Genoa, Italy Via Pastore, 1-16132 Genoa, Italy; E-Mails: am.spagnolo@unige.it (A.M.S.); gianluca.ottria@unige.it (G.O.); perdelli@unige.it (F.P.)

**Keywords:** underground railway, airborne particulate matter, metallic components, ROS

## Abstract

*Background*: Exposure to the particulate matter produced in underground railway systems is arousing increasing scientific interest because of its health effects. The aim of our study was to evaluate the airborne concentrations of PM_10_ and three sub-fractions of PM_2.5_ in an underground railway system environment in proximity to platforms and in underground commercial areas within the system, and to compare these with the outdoor airborne concentrations. We also evaluated the metal components, the cytotoxic properties of the various fractions of particulate matter (PM) and their capacity to induce oxidative stress. *Method*: We collected the coarse fraction (5–10 µm) and the fine fractions (1–2.5 µm; 0.5–1 µm; 0.25–0.5 µm). Chemical characterisation was determined by means of spectrometry. Cytotoxicity and oxidative stress were evaluated by 3-(4,5-dimethylthiazol-2-yl)-2,5-diphenyltetrazolium bromide (MTT) assay and Reactive Oxygen Species (ROS) assessment. *Results*: The concentrations of both PM_10_ and PM_2.5_ proved to be similar at the three sampling sites. Iron and other transition metals displayed a greater concentration at the subway platform than at the other two sites. The 2.5–10 µm and 1–2.5 µm fractions of PM from all three sampling sites determined a greater increase in ROS; the intensity of oxidative stress progressively declined as particle diameter diminished. Moreover, ROS concentrations were correlated with the concentrations of some transition metals, namely Mn, Cr, Ti, Fe, Cu, Zn, Ni and Mo. All particulate matter fractions displayed lower or similar ROS values between platform level and the outdoor air. *Conclusions*: The present study revealed that the underground railway environment at platform level, although containing higher concentrations of some particularly reactive metallic species, did not display higher cytotoxicity and oxidative stress levels than the outdoor air.

## 1. Introduction

Several epidemiological studies have demonstrated that airborne particulate matter is responsible for serious health effects, especially in urban areas. Specifically, fine and ultrafine particles are associated with acute and long-term effects such as cancer and cardiovascular disease [1,2,3].

A report from the prospective Cancer Prevention II study of the American Cancer Society, involving about 500,000 participants, showed that each 10 μg/m^3^ increase in fine particulate matter (PM) air pollution was associated with a 6% increase in all-cause mortality, a 9% increase in the risk of cardiopulmonary mortality and a 14% increase in the risk of lung cancer [4,5]. 

Exposure to the particulate matter produced in underground railway systems is arousing increasing scientific interest on account of the high PM_10_ and PM_2.5_ concentrations reached and their particular chemical composition, which, together with the size, markedly conditions their effect on health [6].

Very high levels of PM have been found in the underground systems of many cities [1,7,8], despite the fact that the trains are electrically powered. In a study by Johansson *et al.* [9] the concentrations of PM_10_ and PM_2.5_ measured over a period of two weeks in the Stockholm underground displayed mean values of 470 and 260 µg/m^3^, respectively. These values were 5- and 10-fold higher than those measured in one of the streets with the heaviest traffic in the center of Stockholm. Moreover, Aarnio *et al.* [10] found that PM_2.5_ levels in underground stations were six times higher than those measured in an urban control environment. In addition, Adams *et al.* [11] evaluated the exposure to PM_2.5_ of commuters who travelled by various means of transport, and found levels of exposure from 3 to 8 times higher among those who used the underground than among those who used surface transport (bicycle, bus, car).

The main chemical constituents of the particulate matter found in underground systems are metals—particularly Fe and Si—and, at lower concentrations, Mn, Co, Ni, Mo, Cd, Cr, Cu, Ca, K [7,10,12,13,14,15,16].

Recently, several studies have aimed to determine the cyto-genotoxic action of underground PM [7,11,14,17,18], which, to date, has chiefly been attributed to the metallic components bound or adsorbed onto particles, [17,19] particularly transition metals, which are able to induce the formation of reactive oxygen species (ROS) [17,20,21]. ROS are involved in a range of mechanisms that cause lipid peroxidation of the membranes and oxidative damage to DNA. Indeed, it has been proposed that oxidative stress may initiate a specific sequence of cellular responses. At lower levels of oxidative stress, antioxidant enzymes are activated in order to protect the lung. If this antioxidant response of the cell fails to provide protection against the generation of ROS, an inflammatory response may be induced to attract inflammatory cells to the site of “injury”. Finally, at toxic levels, cell death occurs through both apoptosis and necrosis [22].

Karlsson *et al.* [23] have shown that the airborne PM present in underground railway systems can cause four times more oxidative stress and eight times more damage to DNA in cells of the respiratory epithelium than the particulate matter found in the outdoor environment. 

To date, no research has been carried out into the metallic composition, the cytotoxic effects and the capacity to induce oxidative stress of sub-fractions of the fine PM present either in proximity to the railway tracks or at other locations, such as commercial premises, inside underground systems. At these latter locations, both shop staff and potential customers spend considerably more time than passengers in transit; they are therefore more exposed to any contaminants that may be present in the air.

The aim of our study was to evaluate the airborne concentrations of PM_10_ and some sub-fractions of PM_2.5_ in the underground railway system environment—not only in proximity to platforms but also in underground commercial areas within the system—of a northern Italian city and to compare these with the concentrations measured outdoors at street level. We also evaluated the metallic component (transition metals and crustal elements), the cytotoxic properties of the various fractions of particulate matter and their capacity to induce oxidative stress.

## 2. Materials and Methods

The underground railway system examined was built in the early 2000s. The station where sampling was conducted handles the highest passenger throughput of the entire underground system considered and its platforms are located about 20 m below street level. The platforms run along both sides of the track and are long enough to accommodate trains made up of three carriages. Some of the carriages are first-generation and have a mixed (electro-dynamic and electro-hydraulic) braking system, while others are second-generation, with a full disc-brake system that is rheostatic up to a speed of 12 Km/h and mechanical at higher speeds. On working days, trains pass through every 3–4 min. The underground commercial premises monitored are located at an intermediate depth between street level and platform level. The outdoor sampling site is situated at street level, on station property just outside the station entrance/exit, and looks out onto a city center street used by traffic. 

### 2.1. Sampling of Airborne Particulate Matter Material 

We conducted preliminary airborne PM monitoring campaigns in the underground environments of the station (platform and commercial-intermediate area) in order to establish the duration of sampling needed to collect enough material for the various analyses. In this preliminary phase, the samples collected underwent only chemical characterisation. Tests were carried out on these samples in order to detect not only metals, but also IPA (benzo(a)pyrene, benzo(e)pyrene, nitropyrene) by means of gas chromatography-mass spectrometry (GC-MS); the latter pollutants, together with cadmium and cobalt, were never detected in these environments and were subsequently eliminated from the panel of parameters to be investigated in the study.

Air sampling was carried out on working days over a period of two weeks by means of samplers made up of a cascade impactor (SKC Inc., Eighty-Four, PA, USA) connected by means of a tube to a battery-driven pump (Leland Legacy^®^ pump, SKC Inc., Eighty-Four, PA, USA). The impactor is equipped with four collector plates able to collect the coarse fraction and the fine fraction (further broken down into three sub-fractions) of the PM, which are deposited on Teflon filters (Fluoropore^©^ membrane Filters, Millipore, Darmstadt, Germany) inserted into the four zones of the impactor. The following particulate matter fractions were separated: 2.5–10 µm (stage A), 1–2.5 µm (stage B), 0.5–1 µm (stage C), and 0.25–0.5 µm (stage D).

During the complete monitoring campaign, the results of which are reported in the present paper, air sampling was carried out simultaneously at the three sites: the underground station platform (Site 1), about 1 m from the track, an intermediate underground area located immediately above Site 1 and utilized for commercial activities (Site 2), and a street-level outdoor environment located above Site 2 (Site 3) on station property just outside the station entrance/exit.

Cumulative sampling was carried out for a total of 50 h at Sites 1 and 2, while in the outdoor urban environment sampling was limited to 31 h, as the filters became clogged over longer times. Air was aspirated at a flow rate of 9 L/min, a total of 27 m^3^ being aspirated at Sites 1 and 2 and 16.74 m^3^ at Site 3. Each filter underwent gravimetric analysis by means of an analytical scale with a precision of ±0.01 mg, in temperature- and relative humidity-controlled conditions. The final concentration of the particulate matter material was obtained by dividing the mass of the particulate matter collected by the volume of air sampled. After weighing, the Teflon filters were cut in half; one half underwent chemical analysis, while the other underwent *in vitro* cytotoxicity testing and ROS determination.

### 2.2. Chemical Analysis: Determination of the Concentrations of Metals 

The coarse and fine particulate matter material collected underwent chemical characterisation. Specifically, some metals known to be typically present in PM in underground systems such as magnesium, potassium, titanium, chromium, aluminium, calcium, manganese, iron, nickel, copper, zinc, molybdenum and barium were sought.

These metals were determined by means of spectrometry, which was preceded by digestion of the sample with hot concentrated acid in order to dissolve the metals associated to the particulate matter. Specifically, the sample was mineralised by adding 4–5 mL of a mixture of nitric acid and hydrogen peroxide (ratio 3:1) in a microwave digester at a maximum temperature of 200 °C for 15 min. The solution yielded by digestion was recovered, filtered through 0.45 µm polyvinyl fluoride (PVDF) filters and brought to a final volume of about 20 mL.

The concentrations of the various metals were determined by means of inductively coupled plasma-optical emission spectrometry (ICP-OES) in the case of Mg, K, Ca, Fe, Mn, Zn and inductively coupled plasma-mass spectrometry (ICP-MS) in the case of Al, Ti, Cr, Ni, Cu, Mo, Ba.

### 2.3. Evaluation of Cytotoxicity and Oxidative Stress 

The following tests were carried out: toxicity assessment, 3-(4,5-dimethylthiazol-2-yl)-2,5-diphenyltetrazolium bromide (MTT) assay and ROS assessment. The samples were tested in triplicate in each experiment.

The portions of the filters assigned to these evaluations had previously been prepared in order to enable the particulate matter to be extracted by means of the technique described by Karlsson *et al.* [23].

#### 2.3.1. Cell Culture

NCI-H727 cells derived from a non-small cell lung carcinoma of a 65-year-old Caucasian woman (ATCC, Rockville, MD, USA) were cultured as monolayers in RPMI (Roswell Park Memorial Institute) medium with 2 mM L-glutamine (Invitrogen Gibco, Milan, Italy) and supplemented with 10% (v/v) fetal calf serum (FCS), 100 IU·mL^−1^ penicillin and 100 μg·mL^−1^ streptomycin. Cells were grown in 25 cm^2^ culture flasks in a humidified 5% CO_2_ atmosphere at 37 °C and were supplied with fresh culture medium every 48 h. When 80%–90% confluence was obtained, the monolayers were subcultured.

#### 2.3.2. Toxicity Assessment

Preliminarily, in order to identify sub-toxic particulate matter doses, *i.e.*, unable to cause the death of over 30% of cells, cytotoxicity was assessed by means of a colorimetric method using a 1% crystal violet solution. To this end, exponentially growing cells were seeded in 96-well microtiter tissue culture plates (1 × 10^4^ cells/well) and incubated in complete medium. After 24 h, the medium was replaced with fresh medium (100 μL) containing 2% FCS and 70 μg/mL of the different PM fractions, using 8 wells per dose. After 3, 6, and 24 h of incubation, the cell monolayers were rinsed three times with phosphate buffered saline (PBS), fixed with 2.5% glutaraldehyde for 10 min, stained with crystal violet, and dried. The dye taken up by the cells was solubilized in dimethyl sulfoxide (DMSO) (100 μL/well), and absorbance was read at 590 nm by using a microtiter plate reader (Multiskan FC M Medical, Milan, Italy). The values obtained at each dose were converted to percentage viability by comparison with a negative control (100%). 

#### 2.3.3. MTT Assay 

Cell viability was assessed by means of the MTT assay, which is based on the reduction of the dye MTT to formazan crystals, an insoluble intracellular blue product, in functional mitochondria of living cells. Briefly, exponentially growing cells that had reached 80% confluence in the monolayer were dissociated and seeded in 96-well microtiter tissue culture plates (1 × 10^4^ cells per well) and incubated in complete medium. After 24 h, the complete medium was replaced with fresh medium (100 μL) containing 2% FCS and PM solutions at a concentration of 70 μg/mL for 6 h. After treatment, the medium was removed and the monolayer was washed with PBS solution (pH 7.4) containing 10 mM D-glucose. The MTT solution (0.5 mg/mL) was added, and the plates were incubated for 3 h at 37 °C in 5% of CO_2_. Subsequently, the supernatant was discarded and 100 µL of DMSO was added to detect MTT reduction.

The absorbance of each well was determined by a microplate reader (Multiskan FC M Medical- Milan) at 570 nm wavelength. Means and standard deviations of absorbance were calculated for each group and referred to sham-treated cells, the value of these latter being considered 100% viability. Results were analyzed by means of one-way ANOVA.

#### 2.3.4. Reactive Oxygen Species Assessment

H727 cells were seeded in 96-well plates at a concentration of 1 × 10^4^ per well and allowed to adhere for at least 18 h. Cells were then washed with PBS and incubated in RPMI with 2% FCS; PM solutions were then added at a concentration of 70 μg/mL and the mixture was left to stand for 3 h. 

In accordance with Shuka *et al.* [24], dichlorofluorescein diacetate (DCFDA) was added after incubation with PM solutions. Specifically, DCFDA at concentration of 20 μM was added to the medium, and incubation was continued for an additional 30 min; cells were then washed with PBS.

The increase in oxidative stress was assessed by fluorometric analysis as depicted by fold changes in MFI (mean fluorescence intensity) values of DCFDA in comparison with the untreated cells. The emission value was recorded at 495 nm Ex and 530 nm Em by means of a fluorescence plate reader (Tecan-Infinite^®^, Männedorf, Switzerland). Emission values were normalized by protein concentration in each well.

### 2.4. Statistical Analysis 

Statistical analysis of the data was performed by means of Stata SE9^TM^ software (Stata, College Station, TX, USA), using the *t*-test, ANOVA and Spearman Correlation test.

## 3. Results 

### 3.1. Concentrations of Airborne Particulate Matter and of the Metallic Component 

**Figure 1:**
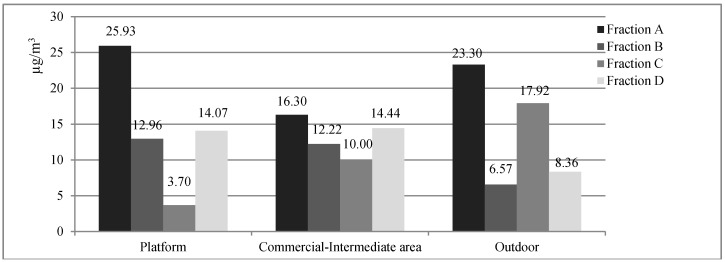
Concentrations (µg/m^3^) of coarse (fraction A: 2.5–10 µm) and fine (fraction B: 1–2.5 µm; fraction C: 0.5–1 µm; fraction D: 0.25–0.5 µm) airborne particulate matter at the three sampling sites.

Figure 1 reports the airborne concentrations of coarse and fine (broken down into sub-fractions) particulates at the three sites considered. Coarse (2.5–10 µm) particulate matter (fraction A) displayed the highest concentrations, particularly at Sites 1 (platform) (25.93 µg/m^3^) and 3 (outdoor environment) (23.30 µg/m^3^).

**Table 1:** Concentrations (ng/m^3^) of transition metals and crustal elements in the various fractions of particulate matter (A, B, C, D) collected at the three sites considered.

Elements		Fraction A (2.5–10 µm) (ng/m^3^)			Fraction B (1–2.5 µm) (ng/m^3^)			Fraction C (0.5–1 µm) (ng/m^3^)			Fraction D (0.25–0.5 µm) (ng/m^3^)		
		Platform	Commercial-intermediate area	Outdoor	Platform	Commercial-intermediate area	Outdoor	Platform	Commercial-intermediate area	Outdoor	Platform	Commercial-intermediate area	Outdoor
**Transition metals**	Cr	15.78	3.11	5.98	3.23	2.07	2.18	1.87	0	1.31	0	0	1.47
	Cu	14.22	13.28	12.41	12.46	6.36	4.64	4.49	2.06	0.74	2.67	6.40	0.86
	Fe	545.34	244.47	117.15	212.01	148.99	115.30	70.79	33.77	30.94	30.54	28.65	32.75
	Mn	8.60	3.57	3.23	2.71	1.94	2.76	1.58	0	0	1.08	0	0
	Mo	10.75	11.00	0	9.19	2.90	0	0	0	0	0	0	0
	Ni	2.96	1.74	0	2.20	1.12	0	0	0	0	0	0	0
	Ti	15.57	4.67	12.44	9.58	2.40	3.73	2.59	1.82	3.25	3.02	1.69	2.81
	Zn	7.73	6.87	15.15	6.98	4.98	6.07	3.49	3.64	5.64	3.01	2.93	5.77
**Crustal elements**	Al	89.14	58.91	67.40	64.73	35.42	39.57	26.53	4.45	5.18	25.03	5.37	2.15
	Ba	122.12	77.75	64.78	95.68	75.82	51.32	98.98	82.42	48.62	99.16	84.54	55.78
	Ca	1568.17	205.33	300.57	256.37	135.70	67.86	57.60	38.22	75.99	30.40	49.19	55.70
	K	26.29	73.62	80.19	10.63	37.43	68.57	6.97	27.31	65.62	5.28	26.54	59.60
	Mg	38.93	23.21	201.81	9.71	12.54	39.36	2.32	3.59	56.44	4.57	0.50	21.47

With regard to the sub-fractions of fine particulate matter, sub-fractions B (1–2.5 µm) and D (0.25–0.5 µm) displayed similar concentrations at Sites 1 and 2 (commercial-intermediate area) and diminished to just above half these levels in the outdoor environment. By contrast, sub-fraction C (0.5–1 µm) was seen to increase from Site 1 to Site 2 and from Site 2 to Site 3.

The concentrations of PM_10_ proved to be 56.66 µg/m^3^ on the station platform, 52.96 µg/m^3^ in the commercial-intermediate area and 56.15 µg/m^3^ in the outdoor environment. Similarly, the concentrations of PM_2.5_ were 30.73 µg/m^3^ on the station platform, 36.66 µg/m^3^ in the commercial-intermediate area and 32.85 µg/m^3^ in the outdoor environment. 

Table 1 shows the chemical determination results. At each monitoring site, the concentration of each of the transition metals considered tended to diminish, more or less markedly according to the metal species, as the size of the particulate matter diminished. Moreover, the coarse fraction of the particulate matter collected at Site 1 (platform) was seen to contain a higher concentration of each metal, except for zinc and molybdenum, than both the coarse particulate matter collected at the other two sites and the fine fractions from the other sites.

Each of the four fractions, with the exception of the smallest (D), displayed a greater concentration of iron at Site 1 than at the other two sites. This trend was particularly evident with regard to the coarse fraction of particulate matter, in which the concentration of iron at Site 1 reached 545.34 ng/m^3^; among all the transition metals considered, this was the highest concentration detected. The iron concentration at Site 2 (commercial-intermediate area) displayed a value that was intermediate between those recorded at Sites 1 and 3. 

With very few exceptions, the “crustal” elements, together with iron, displayed higher concentrations than the other elements analysed; this trend was observed in the various fractions considered, especially the less fine fractions, and at all sites. The element detected at the highest concentration was calcium in the coarse fraction (A) at Site 1 (1568.17 ng/m^3^). This element also displayed a higher concentration than the other elements detected in fraction A at Site 3 (300.57 ng/m^3^), in fraction B at Site 1 (256.37 ng/m^3^) and in fraction C at Site 3 (75.99 ng/m^3^) (Table 1).

### 3.2. Cytotoxic and Oxidative Stress Evaluation 

#### 3.2.1. MTT Assay

**Figure 2:**
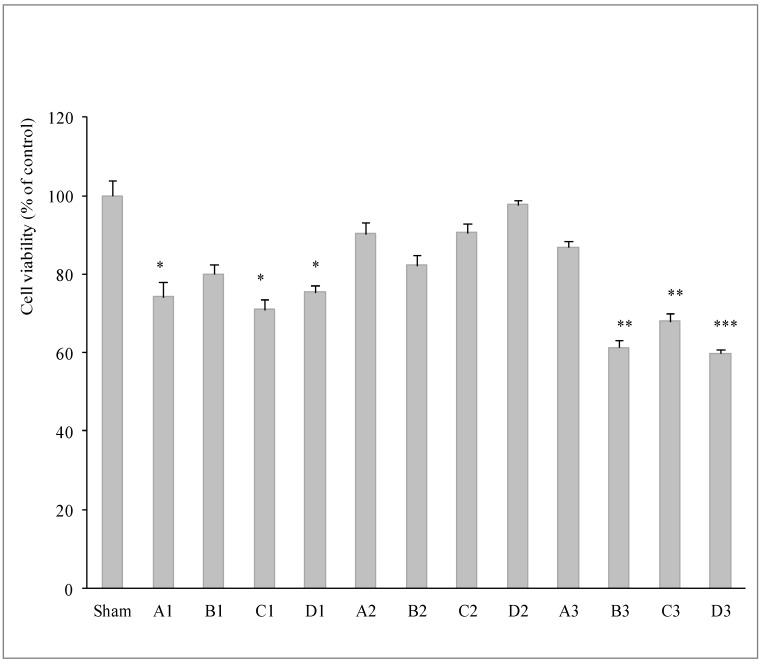
Percentage of viability ± SD in H727 exposed at 70 μg/mL of environmental solutions for 6 h by MTT assay. A = Fraction 2.5–10 µm; B = Fraction 1–2.5 µm; C = Fraction 0.5–1 µm; D = 0.25–0.5 µm. Site 1 = platform; Site 2 = commercial-intermediate area; Site 3 = outdoor. ^*^ *p* < 0.05, ^**^ *p* < 0.01, ^***^ *p* < 0.001 *vs.* untreated cells (Sham).

Figure 2 shows the MTT reduction in H727 cells after 6 h of exposure to the environmental samples. The PM collected at street level was far more cytotoxic than that collected in either the commercial-intermediate area or on the platform. In addition, only the PM components with a diameter less than 2.5 μm were able to determine a statistically significant reduction in cellular activity (*p* < 0.01) in both B3 and C3 samples and a highly significant reduction in D3 (*p* < 0.001). The environmental solution obtained by sampling in Site1 showed a low decrease of viability in all samples tested (*p* < 0.05) apart B1 sample.

No statistically significant difference was observed in cells treated with samples from the commercial- intermediate area, nor in fraction B from samples taken on the platform and in outdoor fraction A.

#### 3.2.2. ROS Analysis

**Figure 3:**
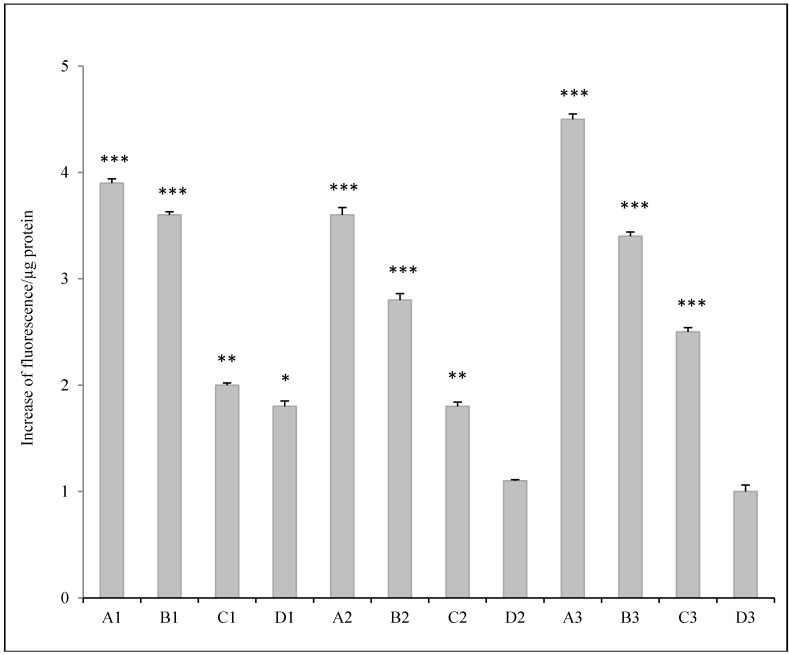
ROS expression in H727 cells treated for 3 h, with different environmental solution. A = Fraction 2.5–10 µm; B = Fraction 1–2.5 µm; C = Fraction 0.5–1 µm; D = 0.25–0.5 µm. Site 1 = platform; Site 2 = commercial-intermediate area; Site 3 = outdoor. ^*^ *p* < 0.05, ^**^ *p* < 0.01, ^***^ *p* < 0.001 *vs.* untreated cells (Sham).

The results of fluorimetric determinations of intracellular ROS production induced by particulate matter solutions are reported in Figure 3. After 3 h of contact with PM solutions, ROS were seen to have increased more markedly in samples treated with fractions A (2.5–10 µm) and B (1–2.5 µm) from all three sampling sites. 

The emission of DCF at the doses tested was approximately 4-fold higher in samples A1, A2, A3 and B1 than in Sham (*p* < 0.001) and approximately 3-fold higher when the cells were exposed to PM solution containing B2 and B3 (*p* < 0.001) in comparison with Sham.

Solutions containing fractions C (0.5–1 µm) and D (0.25–0.5 µm) from Site 1, when inoculated into the cellular substrate, increased DCF emission approximately 2-fold in comparison with Sham (*p* < 0.01). No significant change in fluorescence emission intensity was observed when cells were exposed to PM solutions of PM < 0.25 µm, with the exception of cells treated with solution D1, which increased fluorescence emission intensity 1.8-fold in comparison with Sham (*p* < 0.05). 

From our assessment of the possible correlation between the metals contained in PM and ROS generation, it emerged that the concentrations of transition metals were correlated with increased oxidative capacity. Highly positive correlations were observed between ROS measurements and Mn (rho = 0.8898; *p* < 0.001), Cr (rho = 0.9014, *p* < 0.001), Ti (rho = 0.8531, *p* < 0.001), Fe (rho = 0.8112, *p* < 0.01), Cu (rho = 0.7203, *p* < 0.01) and Zn (rho = 0.7552, *p* < 0.01). Moreover, a consistent correlation was observed with levels of Ni (rho = 0.6033, *p* < 0.05) and Mo (rho = 0.5783, *p* < 0.05). 

## 4. Discussion 

Unlike other indoor environments, underground railway systems display unique features, in that they are enclosed spaces in which particulate matter emissions are particularly abundant, owing to the transit of trains and passengers [25]. These particles tend to accumulate and may reach high concentrations.

Many people spend considerable time in underground railway systems every day. For instance, it has been estimated that in Helsinki about 100,000 people use the underground railway daily and that the average time spent travelling and/or waiting in this environment amounts to about 30 min per person per day [10]. Moreover, many urban railway systems, and indeed underground urban areas in general, host premises for public services and commercial activities, which are frequented by both members of the public and the staff who work in them. 

Various studies, especially in recent years, have been aimed at evaluating pollution due to particulates in underground railway systems [8,26,27] and have documented higher concentrations than in outdoor environments.

In our study, the concentrations of both PM_10_ and PM_2.5_ proved to be similar at the three sampling sites: platform, commercial-intermediate area and street level. Moreover, at Site 1 (platform), these values were much lower than those found in other studies. A possible explanation for this difference lies in the fact that the level of pollution in underground railway systems is influenced by several factors, such as the age of the system (higher concentrations generally being recorded in older stations), the frequency of trains, the braking systems utilized, the daily number of passengers, *etc.* [28].

The particulate matter found in underground railway systems is a complex mixture of particles with high concentrations of Fe and other metals [10]. These metals may originate from outdoor sources and be carried into the underground system by air currents. However, the fact that some metals have been found in higher concentrations in underground environments than outdoors, in various studies [13,29,30] including ours, indicates that some of them are generated *in loco*, e.g., by the friction of the wheels on the rails and the wear of the brakes, *etc.* [29].

Iron in particular is regarded as characteristic of the particulate matter found in underground systems [10,13,31]; in such environments, iron-containing particles are chiefly generated by mechanical wear and tear and friction at the wheel-rail and wheel-brake interfaces and between the pantograph and the overhead cables. These processes initially produce particles of metallic iron, which, on reacting with the oxygen present in the air, are transformed into iron oxide.

In an analysis of PM_10_ in the Rome underground railway system, Ripanucci *et al.* [8] found that iron and silica were the main components of the particulate matter. Moreover, in a study conducted in the London Underground by Seaton *et al.* [7], it emerged that 67% of the PM_2.5_ collected was made up of iron oxides, while quartz and other metals accounted for 1%–2% and the remainder was composed of volatile substances.

In our study, iron was found to be present in greater amounts in the coarse fraction of the particulate matter than in the other fractions (545.34 ng/m^3^ *Vs.* 212.01 ng/m^3^ in fraction B, 70.79 ng/m^3^ in fraction C and 30.54 ng/ m^3^ in fraction D). Moreover, in each of the four fractions, with the exception of the smallest (D), iron displayed a greater concentration at Site 1 (platform) than at the other two sites. Iron also proved to be the most represented of the transition metals sought, not only at the platform level but also at the other sites monitored.

With regard to the underground commercial area situated at an intermediate level between the platform and the street, as mentioned above, the concentration of iron displayed an intermediate value between those recorded at the platform and street levels in all fractions with the exception of D. The same trend was also observed in the total concentration of transition metals.

For what concerns the crustal elements considered (Ca, Ba, Al, Mg, K), the highest concentration recorded was that of calcium in the course fraction A collected at platform level (1568.17 ng/m^3^). As observed in other studies, the calcium detected at platform level could be carried in from outside by air currents. Alternatively, it might originate from construction work inside the underground itself, or from braking systems, *etc.* [14,15]. 

Our results showed that total calcium concentrations (*i.e.*, considering all fractions together) were about 4-fold higher at the level of the platform than at the intermediate-level and outdoor sites. This seems to suggest that this element is mainly produced *in situ*. 

Indeed, particles containing iron and calcium are known to be generated by friction between the brake-pads, which are made of CaCO_3_, and the wheels of the train [14,30].

In order to assess the biological effects of particulate matter, several toxicological studies have recently been carried out [7,17,32]. A study conducted by Janssen *et al.* [33] used various tests, such as DTT assay and ESR assay among others, to determine the oxidative capacity of airborne PM collected at various sites (an underground railway system and several outdoor environments including an area of intense particular traffic). These authors found that the oxidative capacity of PM was far greater in the underground system than at the outdoor sites. 

Similarly, Kam *et al.* [13] recorded a higher level of oxidative stress in particulate matter from an underground railway system than in particulates from an outdoor urban environment (65%) and an overground railway (55%). Metals such as Fe, Ni, Cr and Cd have been shown to display a strong correlation with ROS activity [6,34]. Karlsson *et al.* [23] demonstrated that airborne particulate matter in underground railway systems have a 4-fold greater ability to cause oxidative stress and 8-fold greater genotoxicity than particulates present in outdoor environments, and that this oxidative capacity is essentially due to the redox activity of these metals.

Several epidemiological studies have revealed that exposure to atmospheric PM is a risk factor for various pathologies, including tumours. These effects have chiefly been ascribed to the finest particulate matter (*i.e.*, diameter < 2.5 µm) [35,36]. By contrast, the results of our study indicate that it is the coarse fraction A and sub-fraction B of the fine particulate matter which are associated with the greatest intensity of oxidative stress. Moreover, this intensity progressively declined as particle diameter diminished, as was revealed after the fine particulate matter had been broken down into sub-fractions. 

The declining trend in ROS concentration as particle diameter diminished is similar to the behavior of the majority of the transition metals considered in our study, including those which are recognized as being particularly able to generate free radicals, such as Fe, Mn, Cr and Cu [29,35,37,38,39]. Indeed, the highest concentrations of transition metals—iron, chromium and others—were found in A and B fractions; their abundance would create an optimum condition in which to trigger a Fenton reaction, thus confirming the high oxidative potential of these samples.

Although underground commercial premises in stations are very common, they have, as far as we know, rarely been studied from the cytotoxicity standpoint. From the results of our study, it emerged that all particulate matter fractions, with the exception of fraction D, displayed lower ROS values than the particulates collected at platform level or in the outdoor, street-level environment.

## 5. Conclusions

The present study revealed that the underground railway environment at platform level, although containing higher concentrations of some particularly reactive metallic species (Fe, Cr, Mn, *etc.*), did not display higher levels of cytotoxicity and oxidative stress than the outdoor air. This would seem to suggest that the difference in the concentrations of transition metals between the platform and the outdoor environment was unable to induce a difference in terms of oxidative stress. The concentrations of PM_10_ and PM_2.5_ proved to be similar at the three sampling sites. Moreover, the values recorded at platform level proved to be far lower than those reported in other studies. 

We monitored the commercial-intermediate area because of the long exposure times of staff and customers to any airborne pollutants present at this site. The particulate matter collected was seen to have less ability to induce oxidative stress than that gathered from the other two sites, with the exception of fraction D, which proved comparable to that of the outdoor environment.

As fine particulates were broken down into sub-fractions, we were able to detail their chemical composition and behavior in terms of oxidative stress as a function of size. Moreover, the present research also explored an environment that has rarely been investigated, namely, an underground commercial area.

The results obtained are preliminary and will need to be confirmed by prolonging sampling times, collecting more samples at each site and increasing the number of chemical elements considered. Furthermore, in order to shed further light on the potential healthcare risk to subjects exposed to airborne particulate matter in the underground railway environment, further cytogenotoxicity studies will be required. 
